# The association between dietary zinc intake and risk of pancreatic cancer: a meta-analysis

**DOI:** 10.1042/BSR20170155

**Published:** 2017-06-07

**Authors:** Li Li, Xuesong Gai

**Affiliations:** Department of Emergency Trauma Surgery, The First People’s Hospital of Yunnan Province, Kunming 650032, Yunnan province, China

**Keywords:** Dietary zinc intake, Meta-analysis, Pancreatic cancer

## Abstract

Previous reports have suggested a potential association on dietary zinc intake with the risk of pancreatic cancer. Since the associations between different studies were controversial, we therefore conducted a meta-analysis to reassess the relationship between dietary zinc intake and pancreatic cancer risk. A comprehensive search from the databases of PubMed, Embase, Web of Science, and Medline was performed until January 31, 2017. Relative risk (RR) with 95% confidence intervals (CI) derived by using random effect model was used. Sensitivity analysis and publication bias were conducted. Our meta-analysis was based on seven studies involving 1659 cases, including two prospective cohort studies and five case–control studies. The total RR of pancreatic cancer risk for the highest versus the lowest categories of dietary zinc intake was 0.798 (0.621–0.984), with its significant heterogeneity among studies (*I*^2^=58.2%, *P*=0.026). The average Newcastle–Ottawa scale (NOS) score was 7.29, suggesting a high quality. There was no publication bias in the meta-analysis about dietary zinc intake on the risk of pancreatic cancer. Subgroup analyses showed that dietary zinc intake could reduce the risk of pancreatic cancer in case–control studies and among American populations. In conclusion, we found that highest category of dietary zinc intake can significantly reduce the risk of pancreatic cancer, especially among American populations.

## Introduction

Pancreatic cancer is one of the leading causes of cancer-related mortality worldwide [1–3]. Of the ten leading types of cancer in the United States, pancreatic cancer has a morbidity of approximately 3%, while the total mortality due to pancreatic cancer is approximately 7% [3]. The 5-year survival rate of patients with pancreatic cancer is 6%, and only one-fifth of all patients are eligible for curative surgery at the time of first diagnosis [4]. Therefore, prevention of pancreatic cancer is an important question.

The pancreas is both an endocrine and an exocrine organ. Zinc is involved in a multitude of these processes within the pancreas including glucagon secretion, digestive enzyme activity, and insulin packaging, secretion, and signaling. As a result of this extensive physiological contribution, dysregulation of zinc metabolism may be associated with pancreatic cancer [5]. A published study [6] indicated that higher dietary zinc intake had an increased but not significant association for pancreatic cancer risk. Another two recent large cohorts showed a protection but also nonsignificant association on the risk of pancreatic cancer [7,8]. However, some articles obtained a positive result between dietary zinc intake and pancreatic cancer risk [9,10]. Therefore, the results are not consistent. In this report, we performed a meta-analysis of prospective cohort, case–control studies, or cross-sectional studies on the purpose to investigate the relationship between dietary zinc intake and the risk of pancreatic cancer. We also explored the between-study heterogeneity among studies and publication bias.

## Materials and methods

### Literature search

An electronic search of PubMed, Embase, Web of Science, and Medline was performed until January 31, 2017. The keywords imputed are ‘zinc’ or ‘diet’ or ‘lifestyle’ or ‘Zn’ combined with ‘pancreatic cancer’ or ‘pancreatic carcinoma’ with no language or publication year restriction. The full text of relevant citations from all the results identified has been inspected and analyzed. Relative references in the main outcomes have also been searched and reviewed. The study selection process was performed following the PRISMA [11].

### Selection criteria

Evaluating all the studies above that present quantitative estimates regarding the linkage between dietary zinc intake and pancreatic cancer, and those studies that meet the requirement were embraced in our research and then used for meta-analysis. We made the following strict criteria for our studies: (1) The study design was prospective cohort, case–control, or cross-sectional. (2) Human population studies instead of animals such as mice or rats. (3) The outcome of interest was pancreatic cancer. (4) The independent variable of interest was dietary zinc intake. (5) The risk estimates such as relative risk (RR) or odds ratio (OR) with 95% confidence intervals (CI) were reported or the numbers of case and control and the total numbers were reported. The studies could not satisfy such criteria were ruled out immediately.

### Data extraction and quality assessment

We established a standard data collective form to arrange the data of interest. The data extracted from the studies in use are referring such aspects: author name, year of publication, design of study, sex of population, age, number of cases and participants, value of RR or OR with 95% CI, and relative adjustments. A third reviewer was sought to make a common consensus on the abstracted data. The methodological quality of each study was assessed separately, using Newcastle–Ottawa scale (NOS) [12] to assess the studies, which can be used either as a checklist or as a scale.

### Statistical analysis

The pooled measure was calculated as the inverse variance-weighted mean of the logarithm of RR with 95% CI. A random effects model was used to combine study-specific RR (95% CI), which considers both within-study and between-study variation [13]. Statistical heterogeneity was analyzed using Cochran *I*^2^, which depicts the percentage of variation across studies due to heterogeneity rather than chance [14]. The *I*^2^ was used to assess heterogeneity, and *I*^2^ values of 0, 25, 50, and 75% represent no, low, moderate, and high heterogeneity respectively [15].

Meta-regression with restricted maximum likelihood estimation [16] and subgroup analyses according to study design and ethnicity was performed to assess the potentially important covariates that might exert substantial impact on between-study heterogeneity. Publication bias was analyzed by using Egger’s test and funnel plot [17]. Sensitivity analysis [18] was conducted to describe how robust the pooled estimator was when removing an individual studies at a time. A study was suspected of excessive influence if the point estimate of its omitted analysis lay outside the 95% CI of the combined analysis. STATA version 12.0 (StataCorp LP, College Station, Texas, U.S.A.) was used for the whole meta-analysis. Statistical significance was set at *P*<0.05.

## Results

### Study selection

**Figure 1:**
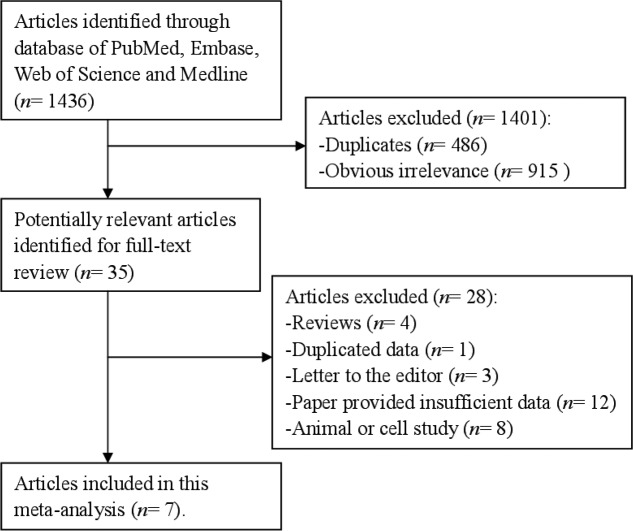
Funnel plot for assessment of publication bias. Study selection process for this meta-analysis.

A flow diagram of the study selection process was showed in Figure 1. Database search led to retrieval of 1436 records from the database of PubMed, Embase, Web of Science, and Medline. There were 486 duplicated records and 915 studies obvious irrelevance when reviewing the abstract and titles that did not meet our demands, which were eliminated from further analyses. After carefully review of the full-text versions of each record, we finally ruled out 28 reports. As a result, 7 references [6–10,19,20] involving 1659 cases were suitable for our meta-analysis.

### Study characteristics

Seven studies were included in our meta-analysis. The characteristics of the included studies on dietary zinc intake and the risk of pancreatic cancer are presented in Table 1. Among them, two were prospective cohort studies and five were case–control studies. Study populations were from four continents: Europe, Asia, America, and Oceania. The results of quality assessment of selected studies were showed in Table 1. Among the seven studies included, all of the studies were in relative high quality (over 6 stars), with the average NOS score was 7.29.

**Table 1:** Characteristics of the included studies on dietary zinc intake and pancreatic cancer risk

Study (year)	Country	Study design	Cases	Participants	Age (years)	RR (95% CI) for highest versus lowest category	Adjustment for covariates	Score quality
Baghurst et al. (1991) [19]	Australia	Case–control	104	357	<50–≥80	0.90 (0.45–1.82)	Adjust for age; pack-years of smoking, tobacco consumption, and vice versa	6
Banim et al. (2013) [7]	U.K.	Prospective	49	23658	40–74	0.82 (0.37–1.81)	Adjusted for age, sex, smoking, diabetes, total energy intake, and body mass index category	8
Bravi et al. (2011) [6]	Italian	Case–control	326	978	34–80	1.39 (0.86–2.24)	Adjusted for age, sex, and center, year of interview, education, tobacco smoking, and history of diabetes, body mass index, and total energy intake	8
Gong et al. (2010) [20]	United States	Case–control	525	2226	21–85	0.89 (0.66–1.2)	Adjusted for age in 5-year groups, sex, and total energy intake, race, education, body mass index, history of diabetes, smoking, physical activity, and alcohol consumption	7
Han et al. (2013) [8]	United States	Prospective	162	77446	50–76	0.94 (0.52–1.71)	Adjusted for age, gender, ethnicity, education, body mass index, physical activity, cigarette smoking status, total alcohol consumption, family history of pancreatic cancer, history of diabetes, and total energy intake	8
Jansen et al. (2013) [10]	United States	Case–control	384	1367	31–92	0.48 (0.32–0.71)	Adjusted for age, sex, cigarette smoking, usual adult body mass index, diabetes mellitus (no, diagnosis <3 years prior or diagnosis 31 years prior), energy intake, number of drinks of alcohol per week, and daily servings of total fruit and vegetable consumption	7
Lin et al. (2005) [9]	Japan	Case–control	109	327	40–79	0.51 (0.28–0.96)	Adjust for age, pack-years of smoking, and energy intake	7

### Association between dietary zinc intake and the risk of pancreatic cancer

**Figure 2:**
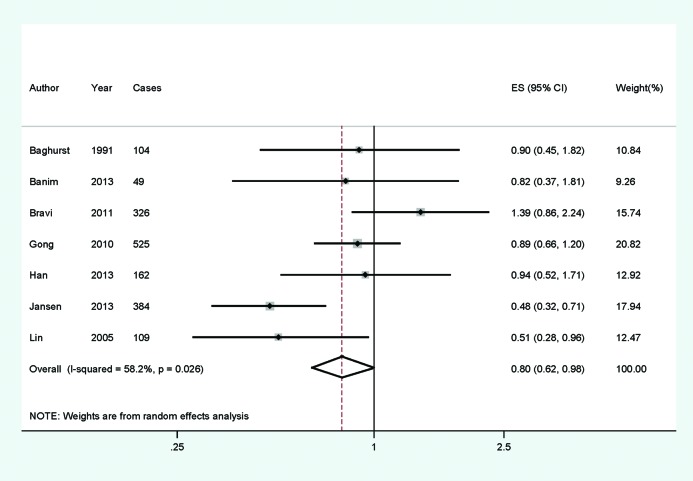
Forest plot for assessment of association between dietary zinc intake and pancreatic cancer risk. Forest plot for assessment of association between dietary zinc intake and pancreatic cancer risk.

The multivariate-adjusted RR of each study of the highest versus the lowest dietary zinc intake is available in Figure 2. The total RR of pancreatic cancer for the highest versus the lowest categories of dietary zinc intake was 0.798 (0.621–0.984), with its significant heterogeneity among studies (*I*^2^=58.2%, *P*=0.026). In order to explore the significant between-study heterogeneity founded in the overall analysis, univariate meta-regression with the covariates of publication year, location (where the study was conducted), and study design (case–control or prospective) was performed. No significant findings were found in the above-mentioned analysis.

**Figure 3:**
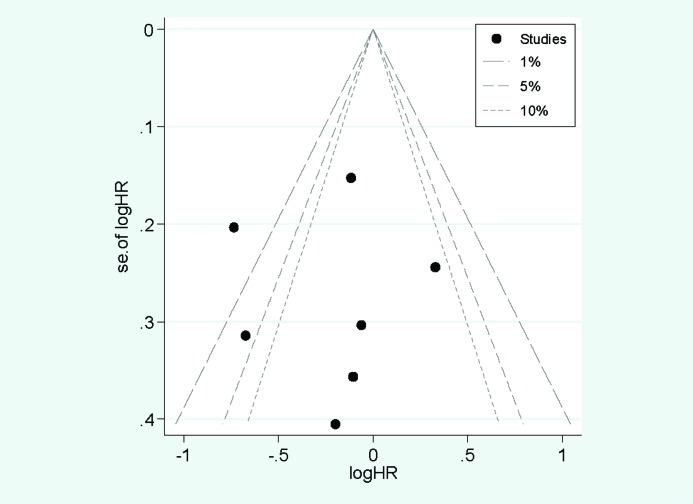
Funnel plot for assessment of publication bias. Funnel plot for assessment of publication bias.

Whether the result of the research has publication bias or not was showed in Figure 3. It showed that all the studies were in a symmetrical distribution. The Egger’s test (*P*=0.997) also showed that there is no publication bias of the meta-analysis about dietary zinc intake and pancreatic cancer.

**Figure 4:**
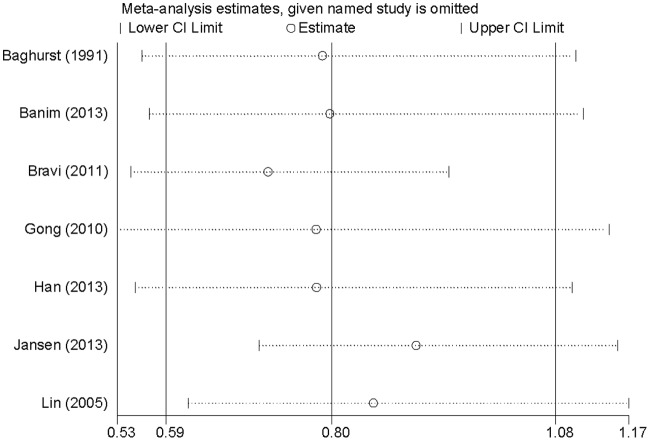
Sensitivity analyses for assessment of association between dietary zinc intake and pancreatic cancer risk. Sensitivity analyses for assessment of association between dietary zinc intake and pancreatic cancer risk.

Sensitivity analysis (Figure 4) showed that no individual study had excessive influence on the association of dietary zinc intake and pancreatic cancer risk when removed one study at a time.

### Subgroup analyses

In this meta-analysis, highest category of dietary zinc intake was reversely associated with the risk of pancreatic cancer. We classified studies into several subgroups for analysis, of which the results were shown in Table 2. Considering the geographical location, the studies that conducted in America (RR=0.729, 95% CI = 0.471–0.987) had statistically significant result. However, the association was not significant either in European populations (RR=1.179, 95% CI = 0.730–1.904) or in mixed populations (RR=0.661, 95% CI = 0.380–1.151). When stratified by study design, case–control studies (RR=0.773, 95% CI = 0.523–0.985) were detected to significantly reduce the risk of pancreatic cancer. But the result was not consistent in prospective cohort studies (RR=0.895, 95% CI = 0.556–1.441).

**Table 2:** Summary risk estimates of the overall and subgroup analyses on dietary zinc intake and pancreatic cancer risk

Subgroups	Number of cases	Number of studies	Risk estimate (95% CI)	Heterogeneity test	
				*I*^2^ (%)	*P*-value
All studies	1659	7	0.798 (0.621–0.984)	58.2	0.026
Study design					
Prospective	211	2	0.895 (0.556–1.441)	0.0	0.787
Case–control	1448	5	0.773 (0.523–0.985)	71.5	0.007
Ethnicity					
American	1071	3	0.729 (0.471–0.987)	69.8	0.037
European	375	2	1.179 (0.730–1.904)	19.7	0.264
Mixed	213	2	0.661 (0.380–1.151)	30.0	0.232

## Discussion

Our study indicated that highest category of dietary zinc intake had significant statistical association on reducing the risk of pancreatic cancer. Those case–control studies and cohort ones were all of high quality. The results in case–control studies and American populations were consistent with the whole result. The total RR of pancreatic cancer for the highest versus the lowest categories of dietary zinc intake was 0.798 (0.621–0.984), with its significant heterogeneity among studies (*I*^2^=58.2%, *P*=0.026).

Diet may be involved in the etiology of pancreatic cancer and dietary variations between countries may explain the differences in incidence of pancreatic cancer. For antioxidants, including dietary zinc intake, there are several plausible biological mechanisms by which they might prevent pancreatic cancer, including inactivating free radicals and reducing oxidative DNA damage, by stimulating immune function and through genetic effects [21,22].

This study reported the relationship between dietary zinc intake and pancreatic cancer risk with a comprehensive meta-analysis involving large number of cases and participants at the first time. The publication bias evaluated by Egger’s test and funnel plot had no significant association for the whole result or subgroup analyses, allowing a much greater possibility of reaching reasonable conclusions between dietary zinc intake and pancreatic cancer risk.

However, evidence of significant between-study heterogeneity was found in the whole result and some subgroups analyses. As we all know, between-study heterogeneity is common in a meta-analysis, and exploring the heterogeneity is necessary in the report [23]. Therefore, we used univariate meta-regression with the covariates of publication year, location (where the study was conducted), and study design (case–control or prospective) to explore the between-study heterogeneity. No significant findings were found in the above-mentioned analysis. We then conducted subgroup analyses by study design and geographic locations to further explore the source of heterogeneity. However, the between-study heterogeneity was evidence in some subgroup analyses.

However, our meta-analysis still had several restrictions. First, as our work embraced seven individual studies that were varied in quality, adjustments, and sample size that can cause the innate influence of our concluded result, but no publication bias was found. Second, due to the unconformity of categories of dietary zinc intake of each study, we did not do the dose–response analysis. Therefore, further studies with detailed category of dietary zinc intake are wanted to assess the dose–response analysis. Third, most studies followed a case–control design that lead to inherent recall and selection bias to retrospective studies. Although different kinds of studies were included, we did subgroup analysis to exclude the interruption. As only 2 prospective cohort studies involving 211 cases were included, more articles with cohort design are wanted.

In summary, the present study suggested that highest category of dietary zinc intake had significant association on reducing the risk of pancreatic cancer, especially among American populations. During some limitation existed in our study, further studies with large cases and participants are wanted to confirm this result.

## Abbreviations

CI: confidence intervals
NOS: Newcastle–Ottawa scale
RR: relative risk
